# Chronic Stress Alters Behavior in the Forced Swim Test and Underlying Neural Activity in Animals Exposed to Alcohol Prenatally: Sex- and Time-Dependent Effects

**DOI:** 10.3389/fnbeh.2018.00042

**Published:** 2018-03-09

**Authors:** Vivian Y. Y. Lam, Charlis Raineki, Lily E. Takeuchi, Linda Ellis, Todd S. Woodward, Joanne Weinberg

**Affiliations:** ^1^Department of Cellular and Physiological Sciences, University of British Columbia, Vancouver, BC, Canada; ^2^Department of Psychiatry, University of British Columbia, Vancouver, BC, Canada; ^3^BC Mental Health and Addictions Research Institute, Vancouver, BC, Canada

**Keywords:** prenatal alcohol exposure, chronic unpredictable stress, medial prefrontal cortex, amygdala, hippocampal formation, paraventricular nucleus of the hypothalamus, *c-fos*, forced swim test

## Abstract

Dysregulation of the hypothalamic-pituitary-adrenal (HPA) stress response has been suggested to play a role in vulnerability to stress-related disorders, such as depression. Prenatal alcohol exposure (PAE) may result in HPA dysregulation, which in turn may predispose individuals to the effects of stress exposure throughout life, and increase their risk of developing depression compared to unexposed individuals. We examined the immediate and delayed effects of chronic unpredictable stress (CUS) in adulthood on behavior of PAE animals in the forced swim test (FST) and the neurocircuitry underlying behavioral, emotional, and stress regulation. Adult male and female offspring from PAE and control conditions were tested for 2 days in the FST, with testing initiated either 1 day (CUS-1; immediate) or 14 days (CUS-14; delayed) post-CUS. Following testing, *c-fos* mRNA expression of the medial prefrontal cortex (mPFC), amygdala, hippocampal formation, and the paraventricular nucleus of the hypothalamus was assessed. Our results indicate that PAE and CUS interact to differentially alter FST behaviors and neural activation of several brain areas in males and females, and effects may depend on whether testing is immediate or delayed post-CUS. PAE males showed decreased time immobile (Day 1 of FST) following immediate testing, while PAE females showed increased time immobile (Day 2 of FST) following delayed testing compared to their respective control counterparts. Moreover, in males, PAE decreased *c-fos* mRNA expression in the lateral and central nuclei of the amygdala in the non-CUS condition, and increased *c-fos* mRNA expression in the CA1 in the CUS-14 condition. By contrast in females, *c-fos* mRNA expression in the Cg1 was decreased in PAE animals (independent of CUS) and decreased in all mPFC subregions in CUS-14 animals (independent of prenatal treatment). Constrained principal component analysis, used to identify neural and behavioral networks, revealed that PAE altered the activation of these networks and modulated the effects of CUS on these networks in a sex- and time-dependent manner. This dysregulation of the neurocircuitry underlying behavioral, emotional and stress regulation, may ultimately contribute to an increased vulnerability to psychopathologies, such as depression, that are often observed following PAE.

## Introduction

Data from both clinical and animal studies suggest that prenatal alcohol exposure (PAE) induces a wide range of adverse neural, cognitive, behavioral, and endocrine effects (Hellemans et al., 2010a; Mooney and Varlinskaya, 2011; Riley et al., 2011; Schneider et al., 2011; Valenzuela et al., 2012; Probyn et al., 2013). Clinically, individuals prenatally exposed to alcohol also often experience mental health problems; in particular, depression and anxiety are disproportionately higher in these individuals than in unexposed individuals (Streissguth et al., 1991, 2004; Famy et al., 1998; O'Connor et al., 2002; O'Connor and Paley, 2009; Pei et al., 2011). Dysregulation of the stress response system, the hypothalamic-pituitary-adrenal (HPA) axis, has been suggested to play a role in vulnerability to stress-related disorders, such as depression and anxiety (Nestler et al., 2002; Jacobson, 2014). As well, programming of the HPA axis by adverse prenatal or early life events may mediate, at least in part, the relationship between the developmental environment and later life susceptibility to stress-related disorders (Phillips et al., 1998, 2000; Matthews, 2000; Meaney et al., 2007; Hellemans et al., 2010a). The fetal HPA axis is highly susceptible to programming by PAE, resulting in altered HPA activity and regulation. Indeed, higher basal and stress cortisol levels have been reported in infants (Ramsay et al., 1996; Jacobson et al., 1999; Haley et al., 2006), and studies using animals have demonstrated increased HPA activation and/or a delayed return to basal levels as well as altered central HPA regulation in PAE compared to control offspring in response to a wide range of stressors (Taylor et al., 1982; Nelson et al., 1986; Weinberg, 1988; Lee et al., 1990, 2000; Redei et al., 1993; Lee and Rivier, 1996; Weinberg et al., 2008). Moreover, individuals prenatally exposed to alcohol are more likely than unexposed individuals to encounter stressful life events (Streissguth et al., 1991, 2004; O'Connor and Paley, 2006). Whether these individuals show higher susceptibility to the consequences of these stressful events is not fully understood. However, it is possible that PAE-induced HPA dysregulation may predispose these individuals to an increased vulnerability to stress-related disorders following subsequent exposure to stressors over the life course.

Using a rat model of PAE, we have previously found that PAE differentially affects males and females in tasks that are suggested to measure depressive-like behaviors—insensitivity to a change in reward value of sucrose (in males), increased immobility in the FST (in females) and altered social interaction (in both males and females)—following chronic unpredictable stress (CUS) in adulthood (Hellemans et al., 2010a,b). We have also shown that these effects of CUS are associated with changes in neural activation and/or mRNA expression of stress neuropeptides in limbic-forebrain regions that are involved in HPA regulation (Herman et al., 2005; Myers et al., 2012)—the medial prefrontal cortex (mPFC), amygdala, hippocampus and hypothalamus (Raineki et al., 2014; Lan et al., 2015). Specifically, in the paraventricular nucleus (PVN) of the hypothalamus, CUS increased corticotropin-releasing hormone (CRH) and arginine vasopressin mRNA expression in PAE compared to C males (Lan et al., 2015), and PAE males failed to show CUS-induced reduction in neural activation in the amygdala and hippocampal formation (Raineki et al., 2014). CUS also decreased basal CRH mRNA expression and neural activation in the mPFC of PAE, but not C, females (Raineki et al., 2014). Additionally, structural and functional abnormalities of these brain areas in humans have been associated with several mental health disorders, including depression (Drevets et al., 2008; Krishnan and Nestler, 2010). Using a neural network framework to investigate the effects of PAE on the neural activity of these areas, we have found that PAE male and female rats activate different neural networks than control animals in response to the elevated plus maze, a stressor that has an emotional or anxiety-related component (Raineki et al., 2014). Moreover, CUS in adulthood differentially affected neural networks in PAE compared to control males and females, suggesting sexually-dimorphic effects of PAE and CUS (Raineki et al., 2014).

In humans, the adverse effects of stress are in some cases experienced immediately after stress exposure but in other cases may not be experienced until days, weeks, or months after the stress exposure has ended. Extensive studies in rats and mice have shown that chronic exposure to mild stressors results in the immediate manifestation of several behaviors related to a depressive phenotype (Willner, 2005) as well as changes in several neurochemical systems (Hill et al., 2012). However, animal studies have also reported delayed effects following chronic exposure to mild stressors or corticosterone, with changes in behavior found at 2 weeks or more following termination of stress exposure, along with concomitant alterations in several neurotrophin-related signaling and heat shock proteins involved in depression (Matuszewich et al., 2007; Gourley et al., 2008a,b). Thus, brain and behavioral consequences of repeated or chronic stress may be temporally dynamic, but in the context of PAE, less is known about the immediate and persistent/delayed effects of stress. Investigation of both the immediate and the persistent/delayed impacts of chronic stress is highly relevant for a more thorough understanding of the etiology of stress-related disorders, such as depression, following PAE.

In the context of PAE, the present study aimed to examine systematically immediate and persistent/delayed effects of adult CUS on behavior of male and female rats in the forced swim test—a test often utilized to measure depressive-like or passive coping behavior—and the neurocircuitry underlying stress and emotional regulation. To determine possible underlying neurobiological changes that may subserve PAE and/or CUS effects, *c-fos* mRNA expression, as a measure of neural activation, was assessed in the mPFC, amygdala, hypothalamus, and hippocampal formation. Because of the multiple brain and behavioral measures, in addition to traditional univariate analyses, further analyses were carried out using Constrained Principal Component Analysis (CPCA), a multivariate exploratory and data reduction technique that allowed us to identify potential neural activation networks associated with interactive effects of PAE and CUS on behavior.

## Materials and methods

### Animals and breeding

All animal use and care procedures were in accordance with the National Institutes of Health Guidelines for the Care and Use of Laboratory Animals and the Canadian Council on Animal Care, and were approved by the University of British Columbia Animal Care Committee. Adult virgin male (275–300 g) and female (265–300 g) Sprague-Dawley rats were obtained from Charles River Laboratories (St. Constant, PQ, Canada). The animal facility was maintained on a 12:12 h light/dark cycle (lights on at 07:00 h), under controlled temperature (21 ± 1°C). Rats were pair-housed by sex and given *ad libitum* access to standard laboratory chow and water while they habituated to the facility for a 7–10 days period. For breeding, males were singly housed, and a female and male were paired. Presence of sperm in vaginal lavage samples taken every morning at 08:00 h indicated day 1 of gestation (GD 1).

### Diets and feeding

On GD 1, females were singly housed in a new colony room in polycarbonate cages on ventilated racks with beta chip bedding. Dams were randomly assigned to one of three treatment groups: (1) Alcohol-fed (PAE; *n* = 13), receiving a liquid ethanol diet with 36% ethanol-derived calories, 6.7% v/v; (2) Pair-fed (PF; *n* = 11), receiving a liquid control diet with maltose-dextrin isocalorically substituted for ethanol, in an amount consumed by a PAE partner (g/kg body weight/day of gestation); and (3) *Ad libitum*-fed control (C; *n* = 10), receiving a pelleted control diet. All diet formulations provided optimal nutrition during pregnancy (Weinberg/Keiver High Protein Ethanol [#710324] and Control [#710109] liquid diets, and Weinberg/Keiver High Protein Pelleted Control Diet [#710109] were prepared by Dyets, Inc., Bethlehem, PA, USA) (Lan et al., 2006).

The diets were presented fresh daily, 1 h prior to lights off to minimize shifts in the maternal corticosterone circadian rhythm (Gallo and Weinberg, 1981), and at that time the volume of liquid diet consumed since the previous night was recorded. All groups also received *ad libitum* access to water. Liquid ethanol diets were introduced gradually over the first 2 days of pregnancy with a 1:2 ratio of liquid ethanol to liquid control diet on GD 1 and a 2:1 ratio on GD 2 to facilitate the transition into a full liquid ethanol diet beginning on GD 3. On GD 17, blood samples were collected from the tail vein 3 h after lights off from a subset of PAE, PF, and C dams (*n* = 3 each) and blood alcohol levels were determined using an assay from Pointe Scientific Inc. (Canton, MI, USA). Experimental diets continued through GD 21. Beginning on GD 22 and throughout lactation, dams received *ad libitum* access to 19% protein laboratory chow (Teklad Global #2019) and water. At birth (postnatal day, PND 1), pups were weighed and litters randomly culled to 12 (6 males, 6 females when possible). If necessary to maintain litter size, pups born on the same day, from the same prenatal treatment group, were fostered into a litter. Dams and offspring were weighed weekly but were otherwise undisturbed until weaning on PND 22, after which pups were group housed by litter and sex until they were pair-housed at ~PND 40. Animals of the same sex and from the same prenatal treatment, but from different litters born ± 2 days apart were paired. Weaned pups and adults were fed an 18% protein chow (Teklad Global #2018) and housed on standard non-ventilated racks.

A cohort of offspring that received the same prenatal treatment was left undisturbed, except for weekly cage changing, until termination.

### Chronic unpredictable stress (CUS) paradigm

Animals from each experimental group were randomly assigned to either stress (CUS) or no-stress (non-CUS) conditions. CUS involved 10 days of twice daily unpredictable exposure to mild stressors at variable time and order, with a minimum of 2 h between stressors; by the end of the 10-day period, all CUS rats experienced each stressor the same number of times. This is a modified CUS protocol, shorter in duration than that described in the literature (Willner et al., 1987; Hill et al., 2012) and designed to produce primarily psychological stress. This is based on findings that robust and reliable effects of CUS have been suggested to present as early as 10 days following the onset of stress exposure (Hill et al., 2012), and that the HPA axis of PAE animals tend to be hyperresponsive to stressors (Hellemans et al., 2010a); therefore, a shorter 10-day model was employed to avoid potential ceiling effects in PAE subjects. Stressors used included: Platform: Animals individually placed on 20 × 20 cm transparent Plexiglas platforms elevated at a height of 90 cm. Restraint: Animals individually restrained in PVC tubes (15 × 6 cm for females and 19 × 7 cm for males) with ventilation holes for 30 min. Soiled Cage: Pairs of cage mates put in a cage with soiled bedding from other animals for 1 h. Wet Cage: Pairs of cage mates put in an empty cage containing 1 cm of room-temperature water at the bottom for 1 h, without access to food or water. Social isolation: 12 h of isolation beginning at lights off without food and water, followed by 1 h of water deprivation in the home cage in the morning; Wet bedding: Just before lights off, 400 ml water at room temperature was poured onto the bedding of the home cage. Animals were left for 13 h beginning at lights off on damp bedding and given a clean cage at the end. Body weights were measured both on Day 1 and on the day following termination of CUS.

### Behavioral testing

Behavioral testing began 1 day or 14 days after the end of CUS (CUS-1 and CUS-14, respectively). Habituation to the testing room occurred 24 h prior to each behavioral test. All animals were assessed on consecutive days, with a one-day break between tests, in the open field, elevated plus maze, light-dark emergence, and forced swim (FST) tests. This paper will focus on the behavior from the FST.

The FST apparatus was a transparent Plexiglas cylinder (20 cm diameter, 60.5 cm height). The cylinders were filled with 25 ± 1°C water to a 44.5 cm depth to prevent animals' tails from touching the bottom of the tank (Detke et al., 1995). On Day 1, animals were placed in the cylinders for 15 min to experience the fact that escape was impossible, and on Day 2, they were tested for 5 min. Activity on both days were recorded by a video camera placed directly facing the cylinder for later scoring. Animals were towel-dried after each exposure and returned to preheated home cages. Duration of immobility, swimming, and climbing were measured, following the criteria of Armario et al. (1988); i.e., immobility is when the rat performs the minimum movement necessary to stay afloat, swimming is when the rat moves horizontally around in the cylinder, and climbing is when the forepaws actively break the surface of the water as the rat attempts to climb against the walls.

Testing occurred under dim lighting with white noise (30 dB) in the background to dampen random noise during the light phase of the circadian cycle. Behaviors in the FST were recorded and scored using Observer 5.0 software (Noldus, Wageningen, The Netherlands). All behaviors were analyzed by an observer blind to the prenatal treatment and CUS conditions. For the FST, *n* = 7–8/prenatal treatment/CUS condition/sex.

### Tissue collection

Whole brains of the cohort of animals that was left undisturbed since being paired as cage mates were collected following decapitation under basal conditions. That is, the brains were collected in less than 3 min from touching their cages. For all other animals, whole brains were collected following decapitation 30 min after the onset of testing on Day 2, based on time-course studies demonstrating that *c-fos* mRNA expression generally peak at 30 min (Cullinan et al., 1995; Tang et al., 1997). Others have also successfully measured *c-fos* mRNA at the 30-min time point (da Costa et al., 1996; Torres et al., 1998; Ons et al., 2004; Kearns and Spencer, 2013). Whole brains were snap frozen on powdered dry ice and stored at −80°C. The brains of the 6 animals with the highest time immobile per prenatal treatment/CUS condition/sex were processed for *in situ* hybridization.

### *In situ* hybridization

Twenty micrometer coronal sections were collected on a cryostat at −16^o^C, mounted on slides (Superfrost slides, Fisher Scientific), and stored at −80°C. For all brain areas, *n* = 4–6/prenatal treatment/CUS condition/sex. Samples from all animals for each brain region were processed simultaneously.

#### Probe and labeling

A ribonucleotide probe was used to detect *c-fos* mRNA in the medial prefrontal cortex [mPFC; anterior cingulate (Cg1), and prelimbic (PrL) and infralimbic (IL) cortices], amygdala (central, medial, lateral, and basal nuclei), paraventricular nucleus of the hypothalamus [medial parvocellular dorsal division (mpdPVN)], and the hippocampal formation [dentate gyrus (DG), CA3, CA1, and ventral subiculum]. The rat *c-fos* ribonucleotide probe was prepared using a 2,116 bp template provided by Dr. Victor Viau (Department of Cellular and Physiological Sciences, The University of British Columbia, Canada). The ribonucleotide probe was labeled with 35S-UTP (Amersham Biosciences, NJ, USA) using Polymerase T7 and Promega Riboprobe System (Promega Corporation, Madison, WI, USA). Micro Bio-Spin 30 Columns (Bio-Rad, CA, USA) was used to purify all probes. Oxidation was prevented by the addition of 1M of DTT.

#### Hybridization

Thawed slides (20 min) were fixed in formalin (30 min), and then pre-hybridized as previously described (Raineki et al., 2014). Briefly, slides underwent a series of washes, dehydrated through a series of increasing concentration of ethanol, delipidated in chloroform, and finally air-dried. After hybridization buffer mixed with the probe (activity of 1 × 10^6^ cpm/slide) was applied, slides were covered with HybriSlips (Sigma-Aldrich, ON, Canada). Following incubation overnight at 55°C in humidified chambers (75% formamide). HybriSlips were removed and slides were rinsed through a series of washes, dehydrated through a series of increasing concentration of ethanol, and air dried overnight.

Kodak BioMax MR autoradiography film was exposed to hybridized slides of the medial prefrontal cortex (mPFC) and hippocampal formation. Exposure times were as follows: 6 days for the mPFC, and 28 days for the hippocampal formation. The exposed autoradiographic films were developed using Kodak GBX developer and fixer.

Hybridized slides for the mpdPVN and the amygdala were dipped in Kodak NTB2 autoradiography emulsion (diluted 50:50 with distilled water) and exposed in desiccated, sealed, and light-tight boxes (4°C) for 86 days in the PVN, and 134 days in the amygdala. Slides were developed using Kodak D19 developer and standard Kodak fixer, counterstained with 0.1% Walpole Toluidine Blue, and coverslipped with Permount (Fisher Scientific Ltd.).

### Densitometric analysis

The autoradiographic films for mPFC and hippocampal formation were scanned and analyzed using Scion Image 4.0.3.2 (National Institutes of Health, USA) according to the Paxinos and Watson Stereotaxic Rat Brain Atlas, Fifth edition (Paxinos and Watson, 2004). Two sections from the left hemisphere and two from the right hemisphere for each brain subregion were traced free-hand, the standard method in our laboratory, resulting in a total of four measurements that were acquired from each subregion of each animal. Background was measured on the same side from the forceps minor for the mPFC and corpus callosum for the hippocampal formation. Corrected mean gray values were obtained by subtracting the background level from each of the four measurements and the four measurements from each animal were then averaged together for analysis.

*In situ* signals on the emulsion-dipped slides for PVN and amygdala were visualized with a Q-imaging monochrome 12-bit camera attached to a Zeiss Axioskop 2 motorized plus microscope. Images were captured under dark field illumination using Northern Eclipse v6.0 (Empix Imaging, Inc., Mississauga, ON, Canada) and analyzed with ImageJ 10.3 software (National Institutes of Health, USA) according to the Paxinos and Watson Stereotaxic Rat Brain Atlas, Fifth edition (Paxinos and Watson, 2004). Two sections from the left hemisphere and two from the right hemisphere for each brain subregion were traced–with a fixed circle (diameter: 0.2 mm; scale 496 pixels/mm) for the mpdPVN or free-hand for the amygdala–for a total of four measurements that were acquired from each subregion of each animal. Background was measured, on the same side, from the adjacent internal capsule for the PVN and optic tract for the amygdala. Corrected mean integrated density was obtained by subtracting the background from each of the four measurements and these were then averaged together for analysis.

### Statistical analyses

Univariate analyses of variance (ANOVAs) were performed using IBM Statistical Package for the Social Sciences (SPSS) Statistics 20 software (IBM, Armonk, NY, USA) for the factors of prenatal treatment, CUS condition, and sex. Because main or interactive effects of sex were revealed for the behavioral measures, all ANOVAs were then run separately for males and females. Significant main effects (e.g., effects of prenatal treatment independent of CUS, and effects of CUS independent of prenatal treatment) and interactions (prenatal treatment × CUS exposure) were then examined using *post-hoc* pairwise comparisons with Šídák correction. Differences were considered significant at *p* ≤ 0.05.

Developmental data were analyzed using repeated-measures ANOVA (RM-ANOVA), with prenatal treatments (C, PF, PAE) as the between-subjects factor, and day of gestation or lactation for the dams and postnatal day for the offspring as the within-subjects factor. A separate one-way ANOVA was used to analyze pup body weights on PND 1. For all developmental data, degrees of freedom were adjusted using the Greenhouse-Geisser estimates of sphericity.

Change scores of body weight (i.e., post-CUS minus pre-CUS body weight of the same animal) were analyzed by ANOVAs for the factors of prenatal treatments and CUS exposure (Non-CUS, CUS).

Behavioral and *c-fos* mRNA data were analyzed using ANOVAs for the factors of prenatal treatment and CUS exposure (Non-CUS, CUS-1, CUS-14). Further analyses utilized planned comparisons to test the *a priori* hypotheses that: (1) PAE will alter depressive-like behavior and neural activation in response to behavioral testing; (2a) CUS will differentially alter depressive-like behavior and neural activity in PAE compared to C animals; and (2b) the differential effects of CUS on brain and behavior in PAE compared to C animals may depend on whether testing is immediate or delayed following termination of CUS exposure.

Further analyses using Constrained Principal Component Analysis (CPCA) were carried out to identify networks of brain regions associated with behavioral changes induced by the experimental conditions. CPCA is an exploratory multivariate technique that combines multivariate least-squares multiple regression and principal component analysis (PCA) to allow us to identify potential neural activation networks associated with interactive effects of PAE and CUS on depressive-like behavior. CPCA was performed in two steps, an *external analysis* and an *internal analysis*, and as for the univariate analyses, were run separately for males and females.

In the first step, the *external analysis* uses multivariate multiple regression to separate the overall variance of the data (standardized using z-scores) with multiple dependent variables (i.e., FST behavior on Day 2 and *c-fos* mRNA in multiple brain areas) into what can be predicted by the experimental manipulations (i.e., prenatal treatment and CUS exposure) from what cannot. New independent variables were created using dummy coding. That is, for each rat, “1” was assigned for group membership and “0” for not (Hunter and Takane, 2002). Specifically, three dummy variables were used for prenatal treatment (C, PF, PAE) and three for stress condition (Non-CUS, CUS-1, and CUS-14); 9 dummy variables—3 × 3 (3 dummy variables for prenatal treatment × 3 for stress condition)—were used to examine simple effects of treatment × CUS interaction. This external analysis produced four matrices of predicted scores that reflect the variance in brain and behavioral structures attributable to main effects of prenatal treatment, CUS condition, the overlap between prenatal treatment and CUS condition, and the interaction effect of prenatal treatment × CUS. To test the hypothesis that CUS will differentially alter behavior and neural activity in response to behavioral testing in PAE compared to C animals, only the matrix of the predicted scores of the variance attributable to prenatal treatment × CUS interaction effects was used for analysis in the second step of CPCA. This is the *internal analysis*, which is a PCA, an exploratory data reduction technique that transforms the predicted scores of our dependent variables (i.e., FST behaviors on Day 2 and *c-fos* mRNA) into linear components that explain the maximum amount of total variance. Varimax with Kaiser normalization was used to rotate all PCA solutions–the *component loadings*. To determine the number of components to include, scree plots were inspected (Cattell, 1966; Cattell and Vogelmann, 1977). The component scores for each component were then correlated with the experimental condition to compute *predictor loadings*—which are correlation coefficients—and these were tested for reliable differences from 0 with a *t*-test. Previously published manuscripts provide a more detailed discussion of CPCA (Takane and Shibayama, 1991; Takane and Hunter, 2001; Hunter and Takane, 2002; Woodward et al., 2006, 2013; Metzak et al., 2011; Lavigne et al., 2013, 2015; Takane, 2013; Raineki et al., 2014; Liu et al., 2015; Bodnar et al., 2017; Sanford and Woodward, 2017). CPCA was carried out using MATLAB R2016a (Mathworks, Natick, MA, USA).

## Results

### Developmental data

Blood alcohol levels were 134.1 ± 23.5 mg/dl in PAE dams and undetectable in PF and C dams. On GD 1, there were no differences in body weight among C, PF, and PAE dams, and all dams gained weight during pregnancy {Table 1; main effect of day [*F*_(1.5, 45.8)_ = 1,317.968, *p* < 0.001]}. However, over the course of gestation (GD 7, 14, and 21), C weighed more than both PF and PAE dams {treatment × day interaction [*F*_(3.1, 45.8)_ = 16.277, *p* < 0.001]}. In addition, C weighed more than both PAE and PF dams on lactation day (LD) 1 {treatment × day interaction [*F*_(4.3, 67.4)_ = 14.945, *p* < 0.001]}; however, by LD 8, body weights were no longer different among treatments.

**Table 1:** Dam and offspring body weights (g, mean ± SEM).

**Body weight of dams**	**Treatment during pregnancy**		
	**Control**	**Pair-fed**	**Alcohol-exposed**
Pregnant dams (N)	10	11	13
Maternal death/illness (N)	0	0	0
Perinatal death	0.4 ± 0.2	0.36 ± 0.2	0.38 ± 0.2
Litter size	15.8 ± 0.8	14.7 ± 0.8	15.2 ± 0.7
Total litter mass	104.8 ± 4.4	101.3 ± 4.1	97.9 ± 3.8
**DAM WEIGHT (g)**			
GD 1	287.1 ± 3.5	289.3 ± 3.5	289.5 ± 3.1
GD 7	331.5 ± 4.2	307.5 ± 4.2^a^	305.8 ± 3.7^a^
GD 14	387.8 ± 6.4	354.5 ± 6.4^a^	347.5 ± 5.6^a^
GD 21	497.0 ± 9.2	445.0 ± 9.2^a^	434.8 ± 8.1^a^
LD 1	393.6 ± 7.5	367.0 ± 7.2^a^	349.0 ± 6.6^a^
LD 8	381.4 ± 7.4	369.1 ± 7.1	374.3 ± 6.5
LD 15	378.8 ± 7.4	368.5 ± 7.0	381.4 ± 6.5
LD 22	347.4 ± 6.0	336.3 ± 5.7	350.8 ± 5.3
**Body weight of offspring**	**Prenatal treatment**		
	**Control**	**Pair-fed**	**PAE**
**MALES**			
PND 1	6.9 ± 0.2	7.1 ± 0.2	6.6 ± 0.2
PND 8	17.7 ± 0.6	18.7 ± 0.6	18.0 ± 0.5
PND 15	33.9 ± 2.0	35.3 ± 1.9	33.9 ± 1.8
PND 22	56.1 ± 1.5	57.5 ± 1.4	59.3 ± 1.3
Pre-CUS	548.3 ± 11.2	553.8 ± 11.2	561.4 ± 11.2
**FEMALES**			
PND 1	6.7 ± 0.1	6.8 ± 0.1	6.4 ± 0.1
PND 8	17.1 ± 0.5	17.7 ± 0.5	17.4 ± 0.5
PND 15	33.0 ± 1.3	34.2 ± 1.2	35.2 ± 1.1
PND 22	54.9 ± 1.5	55.7 ± 1.4	56.7 ± 1.3
Pre-CUS	314.2 ± 6.5	311.1 ± 6.5	337.5 ± 6.5^b^

a *PAE = PF < C*.

b *PAE > C = PF*.

There were no effects of prenatal treatment on litter size, total litter mass, perinatal deaths, or birth weights (Table 1).

As expected, both male and female pups gained weight from PND 1 to 22 {Table 1; main effect of day [males: *F*_(1.8, 54.4)_ = 1,358.064, *p* < 0.001; females: *F*_(1.3,40.1)_ = 2,988.417, *p* < 0.001]}, and there were no differences in body weights among treatments during the preweaning period.

### Body weight pre- and post-CUS

On Day 1 of CUS, body weights of adult male offspring did not differ by prenatal treatment. By contrast, adult PAE females weighed more than C and PF females {Table 1; main effect of prenatal treatment [*F*_(2, 69)_ = 4.995, *p* = 0.009]}.

**Figure 1:**
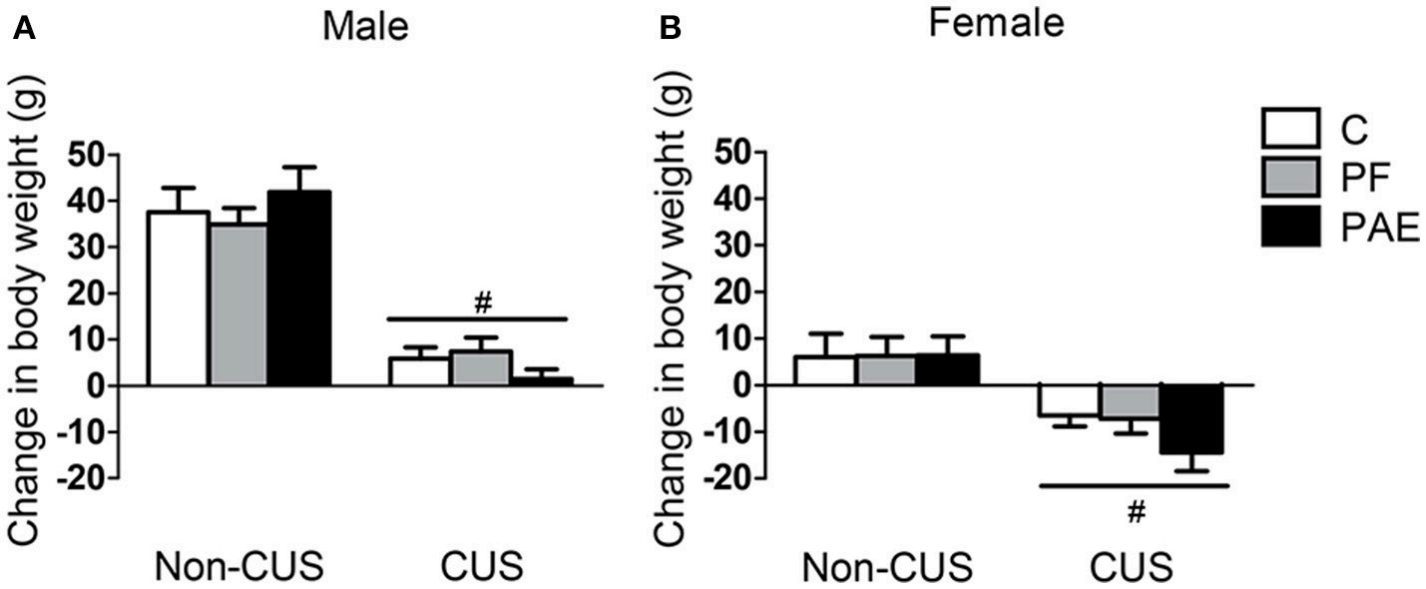
Change in body weight following CUS exposure. Bars represent change scores (difference between pre- and post-CUS body weight; mean ± SEM) following the 10-day period of CUS in control (C), pair-fed (PF), and prenatal alcohol-exposed (PAE) males **(A)** and females **(B)**. ^#^indicates a significant main effect of CUS exposure, where animals exposed to CUS are different from non-CUS animals (non-CUS: *n* = 8/prenatal treatment/sex; CUS: *n* = 16/prenatal treatment/sex).

While all males gained weight over the 10-day period of CUS, CUS males gained less weight than non-CUS males {Figure 1A; main effect of CUS exposure [*F*_(1, 66)_ = 135.729, *p* < 0.001]}. By contrast, non-CUS females gained weight over the 10 days, while CUS females lost weight {Figure 1B; main effect of CUS exposure [*F*_(1, 66)_ = 23.368, *p* < 0.001]}.

### FST

#### Immobility on day 1

**Figure 2:**
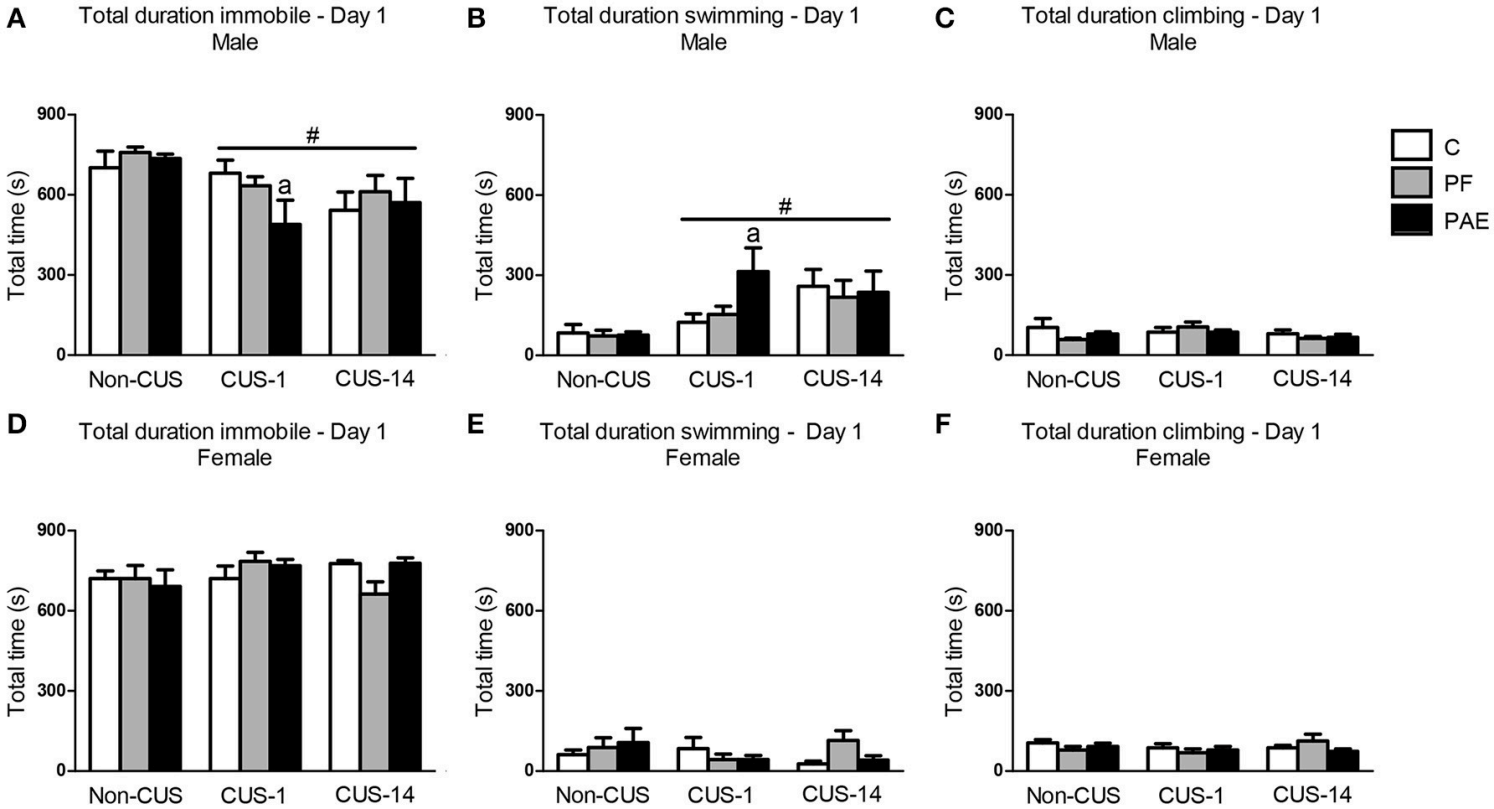
Immediate (CUS-1) and delayed (CUS-14) effects of CUS and prenatal alcohol exposure (PAE) on behaviors in the FST on Day 1. Bars represent the total time (mean ± SEM) control (C), pair-fed (PF), and PAE males and females spent immobile **(A,D)**, swimming **(B,E)**, and climbing **(C,F)** in the FST on Day 1. ^#^Indicates a significant main effect of CUS: CUS-1 and CUS-14 are different from Non-CUS for **(A,B)**; ^a^indicates that CUS-1 PAE is significantly different from CUS-1 C based on *a priori* comparisons (*n* = 7–8/prenatal treatment/CUS condition/sex).

In males, CUS decreased total time spent immobile in the FST regardless of whether testing was immediate (CUS-1) or delayed (CUS-14) {Figure 2A; main effect of CUS [*F*_(2, 63)_ = 5.781, *p* = 0.005]}. Consequently, total time spent swimming in the FST was increased in males under both CUS-1 and CUS-14 conditions compared to males in the non-CUS condition {Figure 2B; main effect of CUS [*F*_(2, 63)_ = 7.345, *p* = 0.001]}. Additionally, a *priori* analyses, based on our hypotheses, showed that CUS-1 PAE males spent less time immobile {Figure 2A; CUS-1 PAE < CUS-1 C (*p* = 0.028)} and more time swimming than CUS-1 C males [Figure 2B; CUS-1 PAE > CUS-1 C (*p* = 0.014)]. There were no effects of prenatal treatment or CUS on climbing (Figure 2C) in males. In females, there were no effects of prenatal treatment or CUS on any behavior (Figures 2D–F).

#### Behavior on day 2

**Figure 3:**
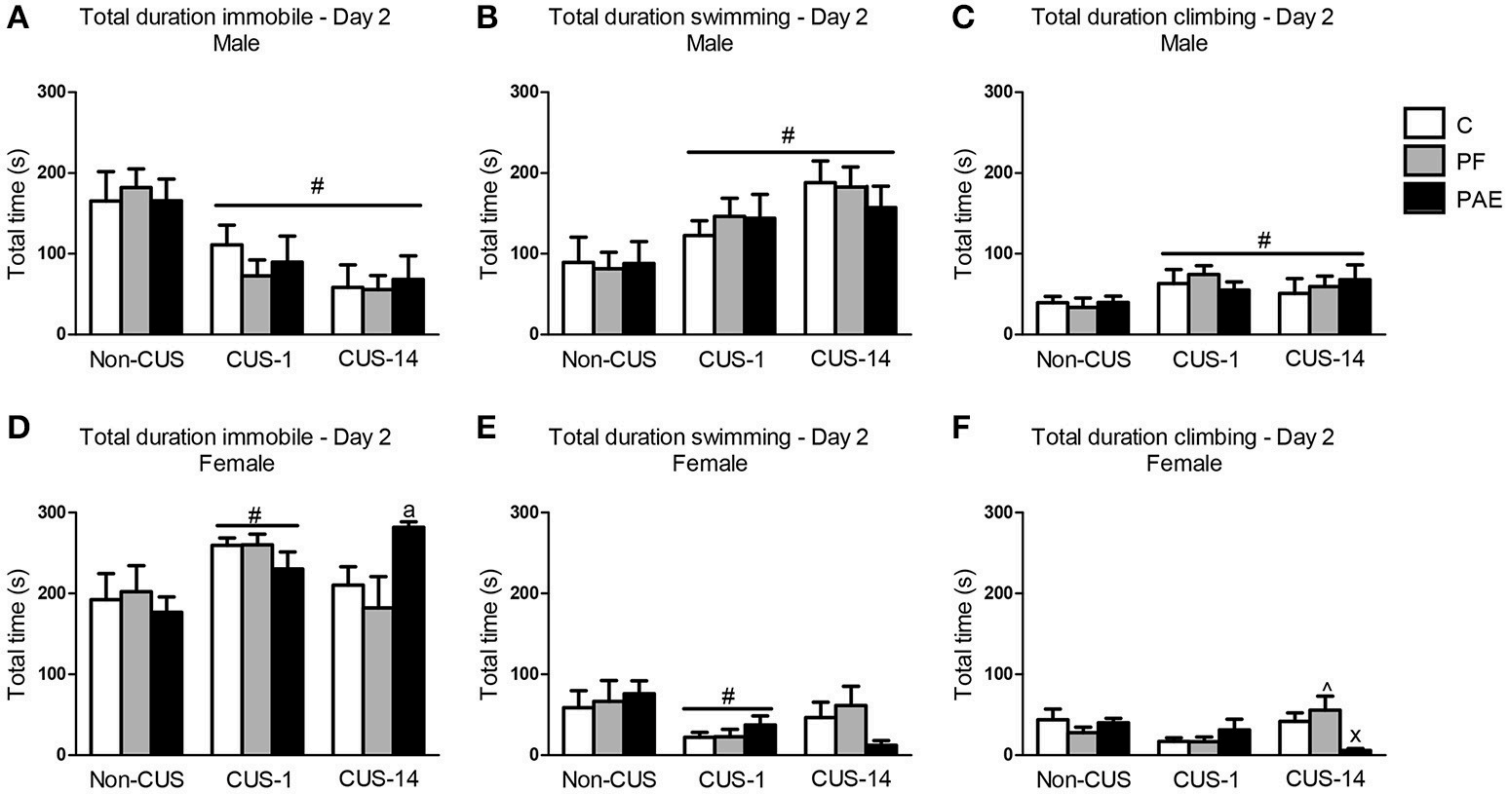
Immediate (CUS-1) and delayed (CUS-14) effects of CUS and prenatal alcohol exposure (PAE) on behaviors in the FST on Day 2. Bars represent the total time (mean ± SEM) control (C), pair-fed (PF), and PAE males and females spent immobile **(A,D)**, swimming **(B,E)**, and climbing **(C,F)** in the FST on Day 2. ^#^indicates a significant main effect of CUS: CUS-1 and CUS-14 are different from Non-CUS for **(A–C)**, and CUS-1 is different from Non-CUS for **(D,E)**; significant treatment × CUS interaction; ^∧^indicates that CUS-14 PF is different from CUS-1 PF; and ^x^indicates that CUS-14 PAE is different from both CUS-14 C and CUS-14 PF; ^a^indicates that CUS-14 PAE is significantly different from CUS-14 C based on *a priori* comparisons; (*n* = 7–8/prenatal treatment/CUS condition/sex).

In males, CUS exposure overall decreased total time spent immobile in the FST {Figure 3A; main effect of CUS [*F*_(2, 63)_ = 13.412, *p* < 0.001]}. Consequently, total duration of swimming and climbing were increased following CUS {Figures 3B,C; main effects of CUS exposure [swimming: *F*_(2, 63)_ = 9.356, *p* < 0.001; climbing: *F*_(2, 63)_ = 3.369, *p* = 0.041]}.

In comparison to males, all females in the CUS-1 condition showed an increase in total time spent immobile compared to non-CUS females {Figure 3D; main effect of CUS [*F*_(2, 61)_ = 4.451, *p* = 0.016]}, and consequently, these females spent less total time swimming {Figure 3E; main effect of CUS [*F*_(2, 61)_ = 4.051, *p* = 0.022]}. By contrast, in the CUS-14 condition, only PAE females spent more total time immobile than C females {Figure 3D; *a priori* analysis: CUS-14 PAE > CUS-14 C (*p* = 0.049)}. CUS-14 PAE females also spent less total time climbing than their C and PF female counterparts, and CUS-14 PF females spent less total time climbing than their CUS-1 counterpart {treatment × CUS interaction [Figure 3F; *F*_(4, 61)_ = 3.450, *p* = 0.013]}.

### *c-fos* mRNA expression

*c-fos* mRNA expression from brains of the cohort of animals collected under basal conditions was undetectable or very low such that measurements could not be reliably performed.

#### Medial prefrontal cortex

**Figure 4:**
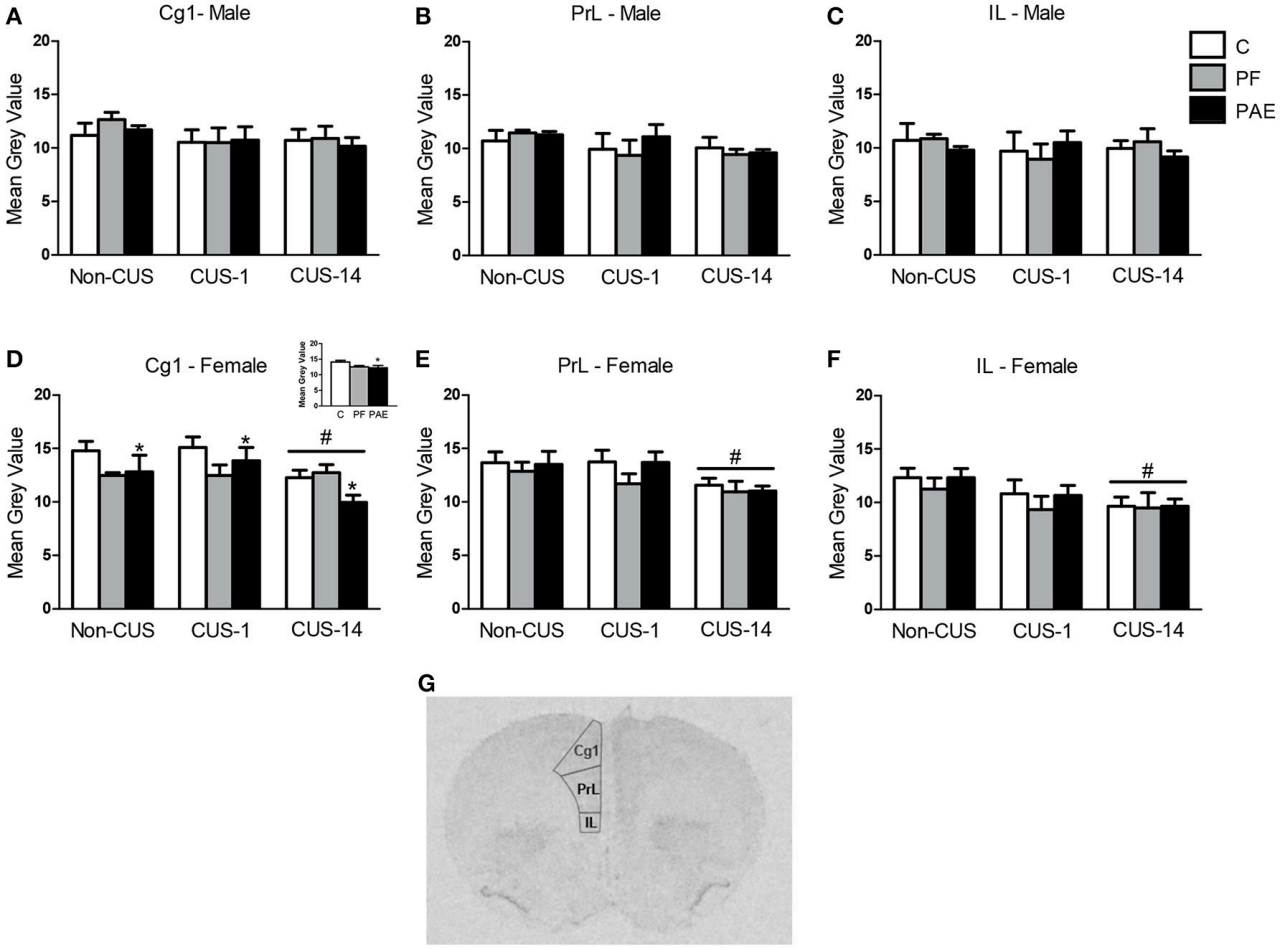
*c-fos* mRNA expression in the medial prefrontal cortex (mPFC) following Day 2 of the FST in response to behavioral testing initiated 1- or 14-day post-CUS (CUS-1 and CUS-14, respectively) in adult male and female control (C), pair-fed (PF), and prenatal alcohol-exposed (PAE) rats. Bars represent mean gray value (mean ± SEM) of *c-fos* mRNA expression in the Cg1 **(A,D)**, PrL **(B,E)**, and IL **(C,F)**. Free-hand delineations of the subregions are demonstrated in a representative image of an autoradiographic film of the mPFC **(G)**. ^*^Indicates a significant main effect of prenatal treatment, where PAE is different from C—see inset graph in **(D)**; ^#^indicates a significant main effect of CUS: CUS-14 is different from Non-CUS and CUS-1 for **(D,E)**; CUS-14 is different from non-CUS for **(F)**; (*n* = 5–7/prenatal treatment/CUS condition/sex).

There were no effects of prenatal treatment or CUS exposure on *c-fos* mRNA expression in the mPFC in males (Figures 4A–C). By contrast, in the Cg1, PAE females overall had lower *c-fos* mRNA expression than their C counterparts {Figure 4D; main effect of prenatal treatment [*F*_(2, 46)_ = 3.335, *p* = 0.044]}. As well, CUS-14 females overall had lower *c-fos* mRNA expression than both CUS-1 and Non-CUS females {Figure 4D; main effect of CUS [*F*_(2, 46)_ = 4.470, *p* = 0.017]}. In the PrL, *c-fos* mRNA expression was lower in CUS-14 females compared to females in the CUS-1 and non-CUS conditions {Figure 4E; main effect of CUS [*F*_(2, 46)_ = 4.893, *p* = 0.012]}. In the IL, *c-fos* mRNA expression was lower in CUS-14 than non-CUS females {Figure 4F; main effect of CUS [*F*_(2, 46)_ = 4.009, *p* = 0.025]}. A representative image of an autoradiographic film of the mPFC is shown in Figure 4G.

#### Amygdala

**Figure 5:**
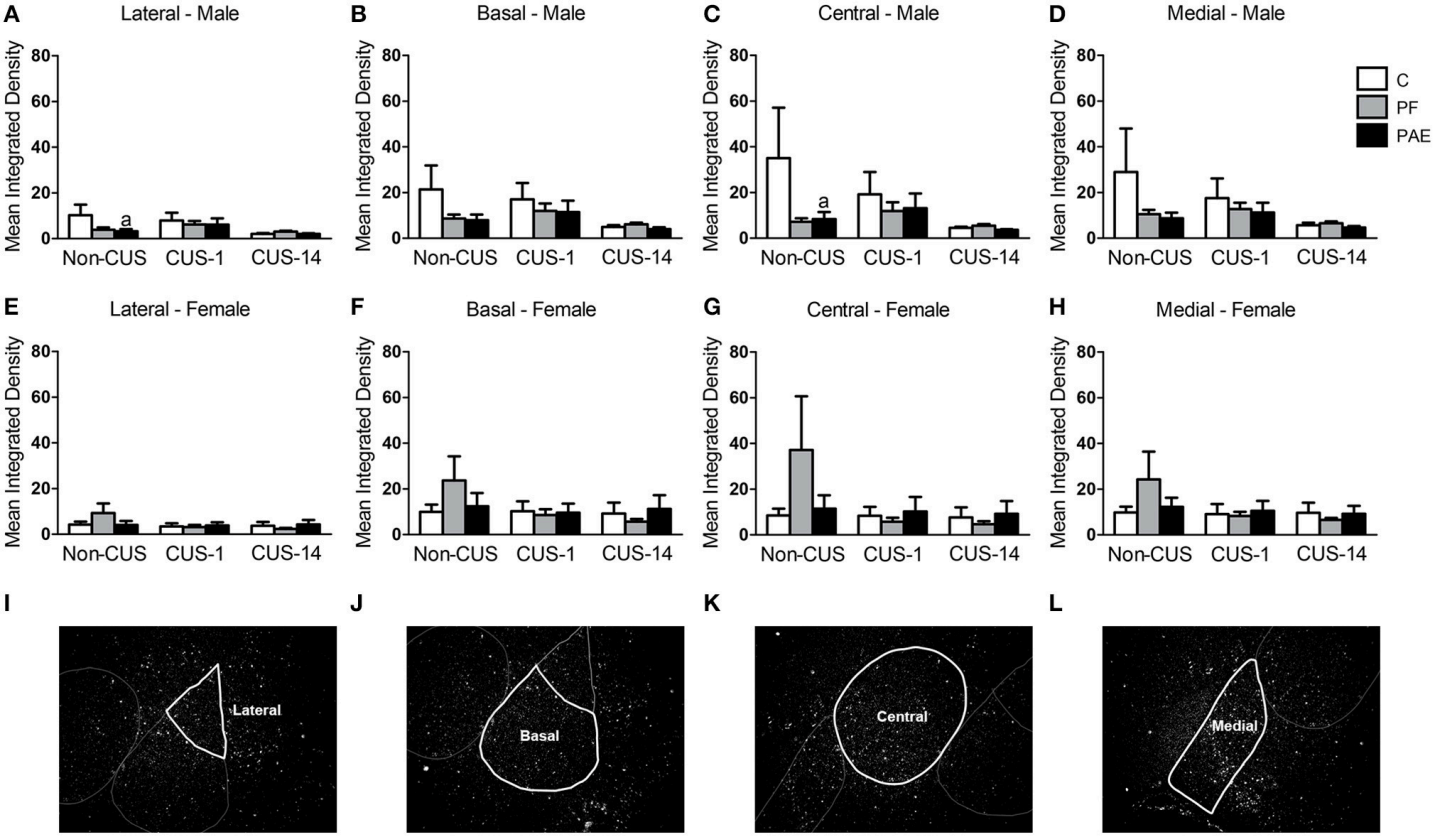
*c-fos* mRNA expression in the amygdala following Day 2 of the FST in response to behavioral testing initiated 1- or 14-day post-CUS (CUS-1 and CUS-14, respectively) in adult male and female control (C), pair-fed (PF), and prenatal alcohol-exposed (PAE) rats. Bars represent mean integrated density (mean ± SEM) of *c-fos* mRNA expression in the lateral **(A,E)**, basal **(B,F)**, central **(C,G)**, and medial **(D,H)** nuclei of the amygdala. Free-hand delineations of the lateral **(I)**, basal **(J)**, central **(K)**, medial **(L)** nuclei are demonstrated in representative images of dark-field photomicrographs of a nuclear emulsion-dipped section of the amygdala. ^a^Indicates that non-CUS PAE is different from non-CUS C based on *a priori* comparisons (*n* = 5–6/prenatal treatment/CUS condition/sex).

In males, ANOVA revealed no effects of prenatal treatment or CUS exposure for any of the amygdala nuclei (Figures 5A–D). However, *a priori* analyses indicated that *c-fos* mRNA expression was lower in Non-CUS PAE males than their C counterparts in both the lateral and central nuclei of the amygdala [Figures 5A,C; Non-CUS PAE < Non-CUS C (lateral: *p* = 0.035 and central: *p* = 0.039)].

In females, there were no effects of prenatal treatment or CUS exposure on *c-fos* mRNA expression in any of the amygdala nuclei (Figures 5E–H). Representative images of dark field photomicrographs of the amygdala are shown in Figures 5I–L.

#### Hippocampal formation

**Figure 6:**
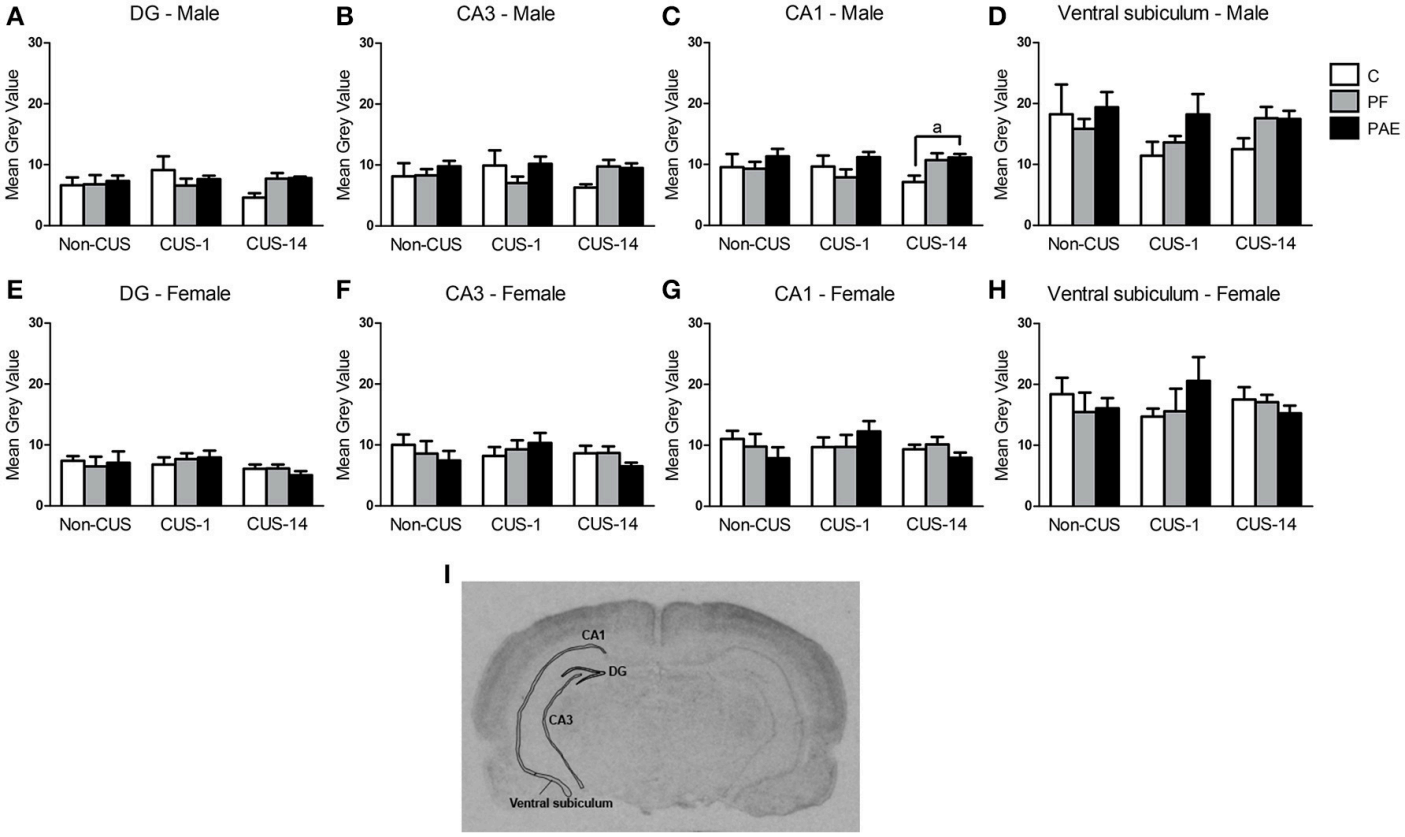
*c-fos* mRNA expression in the hippocampal formation following Day 2 of the FST in response to behavioral testing initiated 1- or 14-day post-CUS (CUS-1 and CUS-14, respectively) in adult male and female control (C), pair-fed (PF), and prenatal alcohol exposed (PAE) rats. Bars represent mean gray value (mean ± SEM) of *c-fos* mRNA expression in the DG **(A,E)**, CA3 **(B,F)**, CA1 **(C,G)**, and ventral subiculum **(D,H)**. Free-hand delineations of the subregions are demonstrated in a representative image of an autoradiographic film of the hippocampal formation **(I)**. ^a^Indicates that CUS-14 PAE is different from CUS-14 C rats based on *a priori* comparisons (*n* = 5–6/prenatal treatment/CUS condition/sex except *n* = 4 for CUS-1 PAE in **(A)**; Non-CUS C, CUS-1 PAE in **(B,C)**; Non-CUS C, CUS-1 C, and CUS-1 PAE in **(D)**; and Non-CUS PF in **(E–H)**.

ANOVA revealed no effects of prenatal treatment or CUS exposure on the CA3, DG, or ventral subiculum in either males or females (Figures 6A,B,D–F,H). However, *a priori* analyses showed that CUS-14 PAE males had higher *c-fos* mRNA expression than their C counterparts in CA1 [Figure 6C; CUS-14 PAE > CUS-14 C (*p* = 0.023)]; inspection of Figure 6C suggests that this was likely due to a directional (non-significant) decrease in CA1 activity in C, but not PAE, males in the CUS-14 compared to the non-CUS condition. There were no effects of prenatal treatment or CUS on CA1 in females (Figure 6G). A representative image of an autoradiographic film of the hippocampal formation is shown in Figure 6I.

#### Hypothalamus

**Figure 7:**
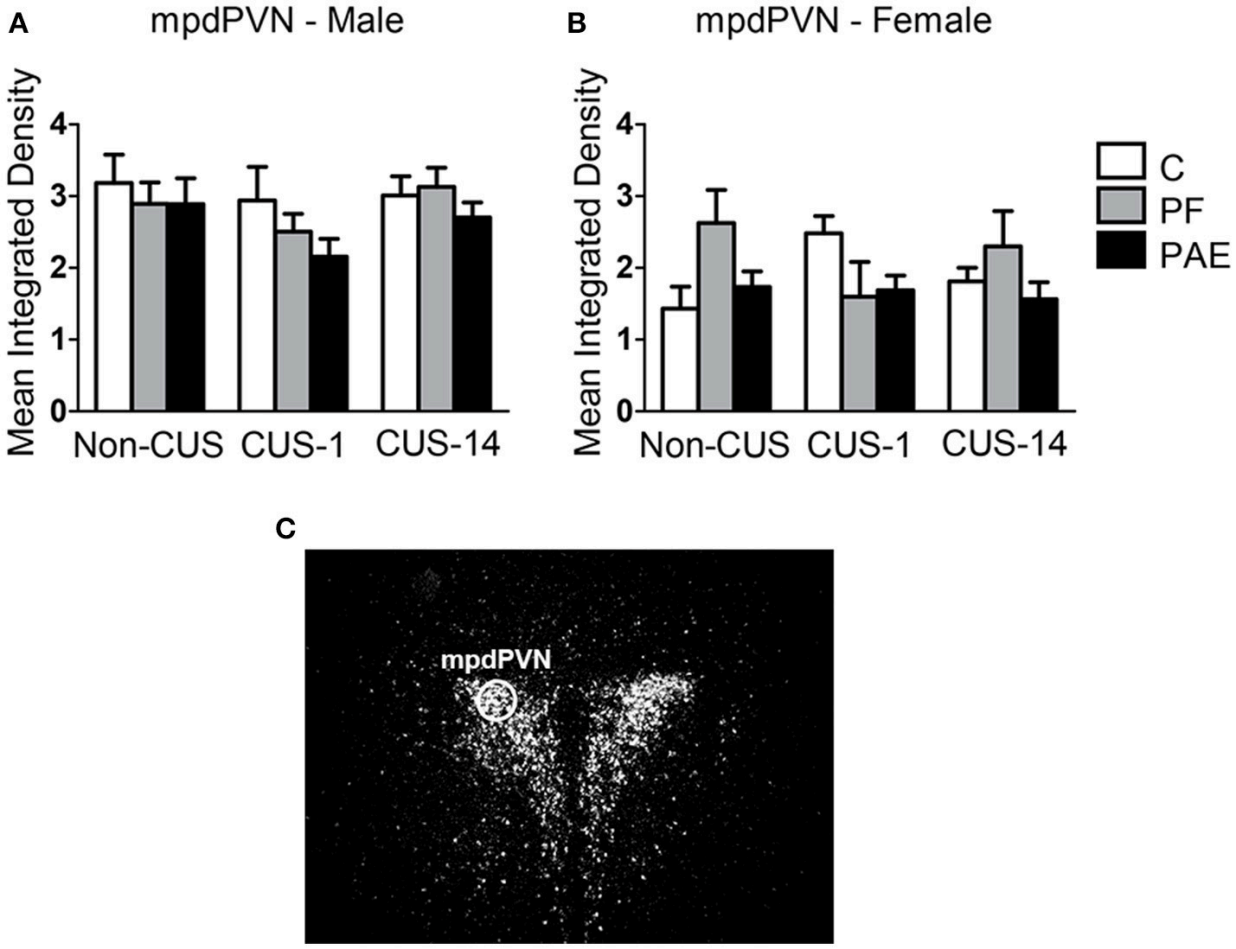
*c-fos* mRNA expression in the mpdPVN following Day 2 of the FST in response to behavioral testing initiated 1- or 14-day post-CUS (CUS-1 and CUS-14, respectively) in adult male and female control (C), pair-fed (PF), and prenatal alcohol exposed (PAE) rats. Bars represent mean integrated density (mean ± SEM) of *c-fos* mRNA expression in males **(A)** and females **(B)**. The mpdPVN was traced using a fixed circle as demonstrated in a representative image of a dark-field photomicrograph of a nuclear emulsion-dipped section of the PVN **(C)** (*n* = 5–6/prenatal treatment/CUS condition/sex).

Neither prenatal treatment nor CUS exposure altered *c-fos* mRNA expression in the mpdPVN in males and females (Figure 7).

### Constrained principal component analysis (CPCA)

Note: The interpretation of the correlations between component scores and experimental group variables (prenatal treatment × CUS condition interaction) depends on the direction of component loadings (i.e., whether it is positive or negative). For example, when *c-fos* mRNA expression of a specific brain subregion positively loads on a component, a higher component score is associated with a higher *c-fos* mRNA expression. A subsequent positive correlation between the component score and the experimental group variable thus indicates that this experimental group is associated with higher *c-fos* mRNA expression from this subregion (i.e., increased activation), whereas a subsequent negative correlation indicates decreased activation. By contrast, when a brain subregion negatively loads on a component, a higher component score is associated with lower *c-fos* mRNA expression. A subsequent positive correlation then indicates decreased activation, whereas a negative correlation indicates increased activation.

#### Males

The effects of prenatal treatment and CUS exposure combined accounted for 19.11% of the overall variance in brain and behavioral structures. Of that, 5.47% was due to the main effect of prenatal treatment independent of CUS, 13.58% was due to the main effect of CUS independent of prenatal treatment, and the remaining 0.06% was due to the overlap between prenatal treatment and CUS. The interaction between prenatal treatment and CUS accounted for an additional 5.37% of the overall variance in brain and behavioral structures. This variance constrained to the interaction between prenatal treatment and CUS was then further analyzed with a PCA, which revealed a four-component solution. The third and fourth components were not further analyzed because their component loadings were below 0.23, which was established as the cut-off point prior to analysis. The first component explained 44.71% of the predictable variance; it was defined as *Medial prefrontal cortex* because the three subregions of the mPFC (i.e., PrL, IL, and Cg1) showed the highest loadings, all in a negative direction. The second component explained 32.78% of the predictable variance, and was defined as *Hippocampal formation*, with the highest positive loadings from all subregions of the hippocampal formation: DG, CA3, CA1 and ventral subiculum. Behavioral measures did not load with brain subregions in either of these components (Table 2).

**Table 2:** Component loadings for the predicted solution in males.

**Variables**	**Medial prefrontal cortex**	**Hippocampal formation**
Cg1	−**0.23**	−0.04
PrL	−**0.33**	−0.08
IL	−**0.38**	0.09
Lateral amygdala	−0.10	0.04
Basal amygdala	−0.09	0.02
Central amygdala	−0.07	0.08
Medial amygdala	−0.08	−0.07
DG	0.07	**0.23**
CA3	−0.03	**0.25**
CA1	−0.09	**0.23**
Ventral subiculum	−0.10	**0.23**
mpdPVN	−0.01	0.07
Immobile duration (Day 2)	0.04	−0.02
Swimming duration (Day 2)	−0.03	0.06
Climbing duration (Day 2	−0.03	−0.08

*Values ≥ 0.23 are set in bold*.

**Figure 8:**
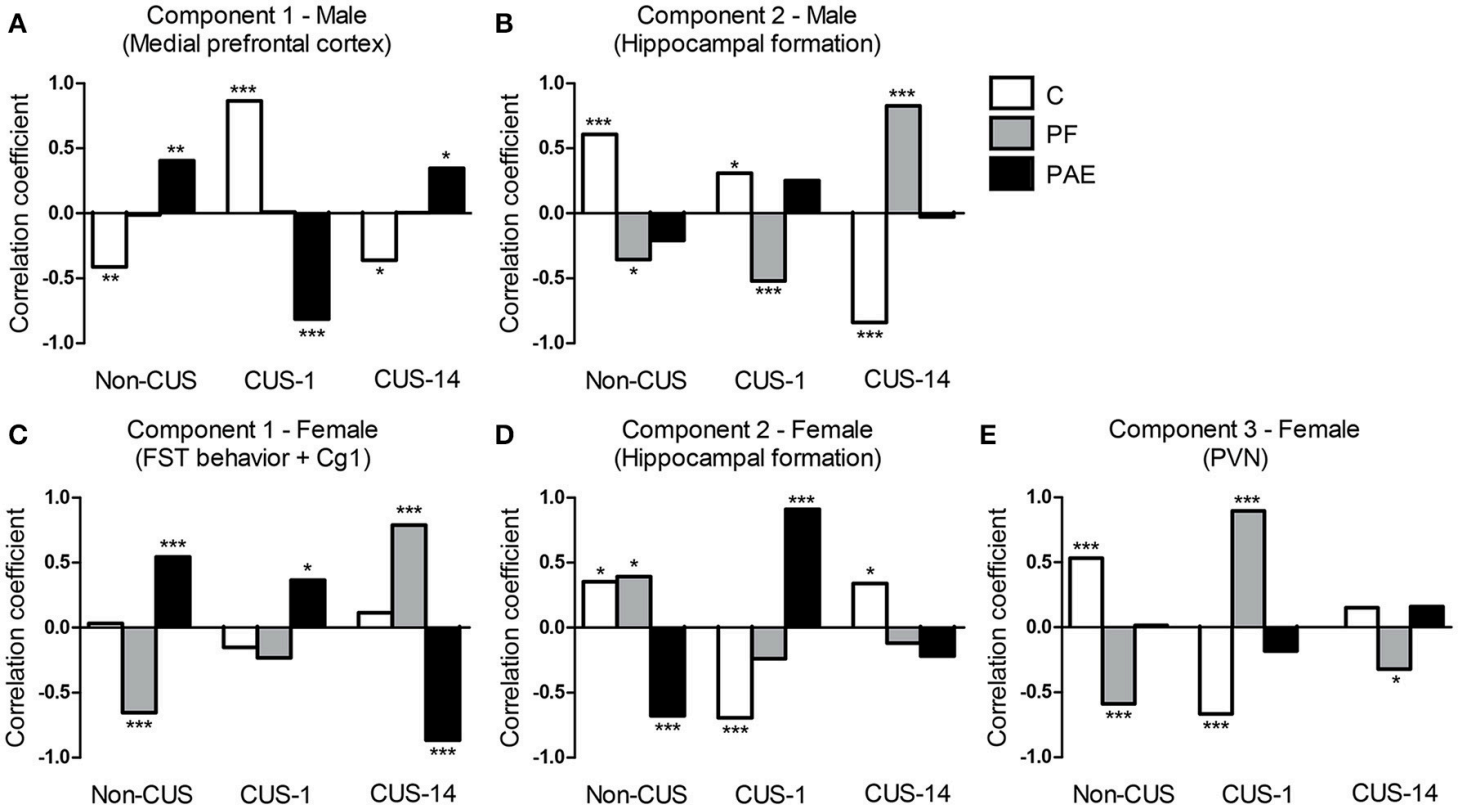
Correlations between the component scores and experimental groups extracted from the constrained principal component analysis. Bars represent the correlation coefficients between component scores and experimental groups (i.e., simple effects of prenatal treatment × CUS exposure interaction) for males **(A,B)** and females **(C–E)**. ^***^p< 0.001; ^**^p < 0.01; ^*^p< 0.05. C, control; PF, pair-fed; and PAE, prenatal alcohol exposed.

For the *Medial prefrontal cortex* network (Figure 8A), correlations between component scores and experimental groups showed opposite patterns for C and PAE animals. For C, there was a negative correlation (increased activation) for the Non-CUS (*r* = −0.41, *p* = 0.005), a positive correlation (decreased activation) for the CUS-1 (*r* = 0.86, *p* < 0.001), and a negative correlation (increased activation) for the CUS-14 condition (*r* = −0.36, *p* = 0.015). By contrast, for PAE, there was a positive correlation (decreased activation) for the Non-CUS (*r* = 0.40, *p* = 0.006), a negative correlation (increased activation) for the CUS-1 (*r* = −0.81, *p* < 0.001), and a positive correlation (decreased activation) for the CUS-14 condition (*r* = 0.35, *p* = 0.020). These opposing patterns suggest that PAE by itself results in differential activation of the *Medial prefrontal cortex* compared to C, and that CUS overall differentially affects PAE and C males. There were no correlations for PF males, regardless of CUS condition.

For the *Hippocampal formation* network (Figure 8B), correlations between component scores and experimental groups showed opposing patterns for C and PF males. For C, there was a positive correlation (increased activation) for both the Non-CUS (*r* = 0.61, *p* < 0.001) and CUS-1 (*r* = 0.31, *p* = 0.039) conditions, but a negative correlation (decreased activation) for the CUS-14 condition (*r* = −0.84, *p* < 0.001). By contrast, for PF, there was a negative correlation (decreased activation) for both the Non-CUS (*r* = −0.36, *p* = 0.016) and CUS-1 (*r* = −0.52, *p* < 0.001) conditions, but a positive correlation (increased activation) for the CUS-14 condition (*r* = 0.83, *p* < 0.001). These opposing patterns suggest that PF by itself results in differential recruitment of the *Hippocampal formation* network compared to C males, and CUS differentially affects PF and C males. There were no correlations for PAE males, regardless of CUS condition.

#### Females

The effects of prenatal treatment and CUS exposure combined accounted for 9.80% of the overall variance in brain and behavioral structures. Of that, 2.55% was due to the main effect of prenatal treatment independent of CUS, 7.18% was due to the main effect of CUS independent of prenatal treatment, and the remaining 0.06% was due to the overlap between prenatal treatment and CUS. The interaction between prenatal treatment and CUS accounted for an additional 10.72% of the overall variance in brain and behavioral structures. This variance constrained to the interaction between prenatal treatment and CUS was then further analyzed with a PCA, which revealed a four-component solution. The fourth component was not further analyzed because its component loadings were below 0.23. The first component explained 50.26% of the predictable variance; it was defined as *FST behavior* + *Cg1* because total duration of immobility, swimming, and climbing from Day 2 in the FST, and Cg1 activity together showed the highest loadings. Duration of immobility loaded negatively, while the other three variables loaded positively in this component. The second component explained 20.94% of the predictable variance, and was defined as the *Hippocampal formation*, with CA1, CA3, and ventral subiculum showing the highest positive loadings. The third component explained 20.12% of the predictable variance, and was defined as *PVN* as the mpdPVN alone loaded negatively on this component (Table 3).

**Table 3:** Component loadings for the predicted solution in females.

**Variables**	**FST behavior + Cg1**	**Hippocampal formation**	**PVN**
Cg1	**0.26**	0.07	−0.15
PrL	0.00	0.08	−0.20
IL	−0.03	−0.07	−0.06
Lateral amygdala	−0.07	−0.07	0.13
Basal amygdala	−0.07	−0.14	0.09
Central amygdala	−0.02	−0.16	0.07
Medial amygdala	0.01	−0.11	0.13
DG	0.10	0.11	0.04
CA3	0.11	**0.26**	−0.01
CA1	0.10	**0.30**	−0.08
Ventral subiculum	0.15	**0.29**	−0.06
mpdPVN	0.01	0.03	−**0.44**
Immobile duration (Day 2)	−**0.51**	−0.04	−0.01
Swimming duration (Day 2)	**0.40**	0.06	0.05
Climbing duration (Day 2)	**0.51**	0.02	−0.06

*Values ≥ 0.23 are set in bold*.

For the *FST behavior* + *Cg1* component (Figure 8C), correlations between component scores and experimental groups showed opposing patterns for PF and PAE females. For PAE, there was a positive correlation (decreased immobility but increased swimming, climbing, and Cg1 activation) for both the Non-CUS (*r* = 0.54, *p* < 0.001) and CUS-1 (*r* = 0.37, *p* = 0.013), and a negative correlation (increased immobility; decreased swimming, climbing, and Cg1 activation) for the CUS-14 condition (*r* = −0.87, *p* < 0.001), whereas for PF, there was a negative correlation (increased immobility; decreased swimming, climbing, and Cg1 activation) for the Non-CUS (*r* = 0.54, *p* < 0.001), no correlation for the CUS-1, and a positive correlation (decreased immobility but increased swimming, climbing, and Cg1 activation) for the CUS-14 condition (*r* = 0.79, *p* < 0.001). These opposing patterns suggest that PAE and PF by themselves result in differential behavior and Cg1 activation compared to C, and both their behavior and Cg1 activity are differentially affected by CUS.

For the *Hippocampal formation* network (Figure 8D), unlike males, correlations between component scores and experimental groups overall showed opposing patterns for C and PAE females. For C, there was a positive correlation (increased activation) for the Non-CUS (*r* = 0.35, *p* = 0.017), a negative correlation (decreased activation) for the CUS-1 (*r* = −0.69, *p* < 0.001), and a positive correlation (increased activation) for the CUS-14 condition (*r* = 0.34, *p* = 0.023). By contrast, for PAE, there was a negative correlation (decreased activation) for the Non-CUS (*r* = −0.68, *p* < 0.001), a positive correlation (increased activation) for the CUS-1 (*r* = 0.91, *p* < 0.001), and no correlation for the CUS-14 condition. As well, for PF females, there was a positive correlation (increased activation) for the Non-CUS (*r* = 0.39, *p* = 0.008), but no correlations for the CUS-1 or CUS-14 conditions in this component. These opposing patterns suggest that PAE by itself results in differential recruitment of the *Hippocampal formation* network compared to C, and CUS overall differentially affects PAE, PF, and C females.

For the *PVN* component (Figure 8E), correlations between component scores and experimental groups showed opposing patterns for C and PF females. For C, there was a positive correlation (decreased activation) for the Non-CUS (*r* = 0.53, *p* < 0.001), a negative correlation (increased activation) for the CUS-1 (*r* = −0.67, *p* < 0.001), and no correlation for the CUS-14 C condition. By contrast, for PF, there was a negative correlation (increased activation) for the Non-CUS (*r* = −0.59, *p* < 0.001) and CUS-14 (*r* = −0.32, *p* = 0.031) conditions, but a positive correlation (decreased activation) for the CUS-1 condition (*r* = 0.90, *p* < 0.001). These patterns suggest that PF by itself causes differential recruitment of the *mpdPVN* compared to C and that CUS overall differentially affects PF and C females. There were no correlations for PAE females, regardless of CUS condition.

## Discussion

Individuals prenatally exposed to alcohol are at a disproportionately higher risk than unexposed individuals of developing mental health problems, with a high incidence of depression reported. Brain areas implicated in the etiology of depression—including, but not limited to, the mPFC, amygdala, hypothalamus, and hippocampal formation—play a multiple role in behavioral, emotional, and stress regulation. The fine coordination of these interconnected brain regions supports appropriate regulation, and dysfunction of any one of these areas could result in maladaptive responses to stressors and/or the emergence of psychopathologies. Our results indicate that PAE and CUS interact to alter behavior in the FST, as well as neural activation of several brain areas, with differential effects in males and females. Our CPCA results suggest that PAE alters the activation of neural and behavioral networks, and CUS differentially affects the networks of PAE, PF and C animals in a sex- and time-dependent manner. Overall, PAE-induced alterations in the neurocircuitry involved in behavioral, stress and emotional regulation, compounded with the impact of the later-life challenge of CUS, may underlie the increased risk for the development of depression in individuals prenatally exposed to alcohol.

### PAE and CUS effects on body weight

Exposure to CUS in adulthood had an adverse impact on body weight regardless of prenatal treatment: males gained less weight while females lost weight compared to their non-CUS counterparts. These results are in line with findings from other studies showing that CUS exposure generally causes reduced weight gain in males (Bielajew et al., 2002; Cox et al., 2011) and are consistent with our previous data demonstrating that CUS may reduce weight *gain* in males and induce weight *loss* in females (Hellemans et al., 2008; Uban et al., 2013). Changes in body weight following CUS also validate the effectiveness of our CUS paradigm. Importantly, our data suggest that males and females may be differentially responsive to the same CUS procedure.

### PAE and CUS effects on FST behavior

There was an overall effect of CUS, independent of prenatal treatment, on behavior in the FST in both males and females, and the effect varied depending on whether testing occurred immediately or following a delay post-CUS. In males, CUS exposure decreased time immobile on both days of FST testing regardless of whether testing was immediate (CUS-1) or delayed (CUS-14), whereas in females, there was an increase in time immobile on Day 2, but only with immediate testing following CUS (CUS-1). The short-term effects of chronic stress on immobility has been variable in the literature: while most report an increase, a few have reported a decrease in immobility in the FST (reviewed in Willner, 2005). These differences may be due to different stress procedures used (e.g., the duration of exposure and types of stressors), the parameters of testing (e.g., the temperature of the water, size of apparatus, and depth of water) (Detke and Lucki, 1996; Slattery and Cryan, 2012), as well as strain of the subjects (Bielajew et al., 2003). Sex of the animal may also influence FST behavior, as we and others have also previously found that chronic stress induces differential alterations in immobility behavior and reward sensitivity to sucrose in males and females (Bielajew et al., 2003; Hellemans et al., 2010a; Dalla et al., 2011; Sachs et al., 2014). We now extend these findings to show that the effects of CUS can also be temporally dynamic depending on the sex of the animal.

Importantly, we found that PAE and CUS interact to differentially alter behavior of males and females in the FST. In males, we found decreased time immobile in PAE compared to C males on Day 1 with immediate testing post-CUS (CUS-1), but no differences between groups on Day 2. By contrast, in females, CUS had no effects on PAE animals on Day 1 of the FST, but had an adverse delayed effect on Day 2: specifically, CUS-14 PAE females showed an increase in time immobile compared to C females. Our previous study showed that PAE males exhibited lower immobility while PAE females exhibited higher immobility than their respective C counterparts in response to immediate testing following CUS, but these effects were observed on Day 2 of testing for males and on both Days 1 and 2 for females (Hellemans et al., 2010b). The difference in results between our previous and current studies may be due to the CUS paradigm used. Nevertheless, our findings suggest differential time-dependent alterations in FST behavior in PAE males and females following CUS.

Immobility in the FST has been suggested to represent “behavioral despair,” which arises after realizing escape is impossible. This stems from the origin of the FST as a preclinical screen for antidepressants; that is, antidepressants that are effective in humans are found to decrease immobility in rats in this task (Borsini and Meli, 1988; Cryan et al., 2005). However, behaviors in the FST can also be described as measures of active-coping strategies, with immobility representing a passive coping response (Slattery and Cryan, 2012; Commons et al., 2017). That CUS selectively increased immobility, whether it is a reflection of behavioral despair or passive coping, in PAE female rats is consistent with clinical literature suggesting that depression may be almost twice as common in women than men, possibly due to increased susceptibility to environmental factors, such as stress, that may trigger depression (Weiss et al., 1999; Kessler, 2003; Altemus, 2006). On the other hand, the decrease in immobility in PAE males may suggest decreased behavioral despair or an increase in active coping. Regardless of interpretation of the meaning of the behavior in the FST, our findings indicate that CUS differentially affected the behavioral response of PAE males and females compared to their control counterparts to the acute stress of FST exposure.

### PAE and CUS effects on neural activation

#### Medial prefrontal cortex

Anatomical and electrophysiological studies suggest that the rat mPFC is related to the primate anterior cingulate cortex (ACC) and dorsolateral PFC (Heidbreder and Groenewegen, 2003; Seamans et al., 2008). The rat mPFC is involved in various aspects of cognitive processing (e.g., working memory, attention, behavioral flexibility, response initiation, motor planning, and reward anticipation), as well as emotional regulation (Heidbreder and Groenewegen, 2003; Seamans et al., 2008; Koenigs and Grafman, 2009). In humans, hypofrontality in the PFC has been associated with depression, with hypoactivation observed in the pregenual ACC—the “affective subdivision” (Vogt et al., 1992; Devinsky et al., 1995; Bush et al., 2000)—and the dorsolateral PFC in individuals with depression (Baxter et al., 1989; Cohen et al., 1989; Martinot et al., 1990; Yazici et al., 1992; Ito et al., 1996; George et al., 1997; Mayberg et al., 1999; Hamilton et al., 2012). We found that in females but not males, PAE caused a reduction in *c-fos* mRNA expression in the Cg1 compared to C and PF, independent of CUS exposure. This PAE-induced hypoactivation is reminiscent of the observed hypofrontality in individuals with major depression, and may therefore subserve an overall predisposition or vulnerability to develop depressive-like behavior.

CUS also resulted in an overall decrease in neural activation in the female Cg1, PrL, and IL, but only with delayed testing post-CUS, regardless of prenatal treatment. Our finding is temporally inconsistent with our behavioral data, but it is possible that while the behavioral consequence of CUS was more apparent immediately, changes in neural activation showed a delayed onset because of potential structural and neurobiological effects of stress that may possibly take time to occur. For example, levels of several neurotrophin-related signaling proteins that are involved with depression, which are decreased in the PFC and hippocampus of suicide patients (Dwivedi et al., 2003, 2009) and of rats following chronic swim stress exposure (Qi et al., 2008), show alterations in hippocampal expression at 2 weeks, but not 1 day following chronic corticosterone (Gourley et al., 2008b). Future studies may help to elucidate the intracellular mechanisms in the mPFC that may underlie the delayed effects of CUS on neural activation. Nevertheless, our findings suggest that PAE females may be more susceptible than C and PF females to the effects of CUS on neural activation such that given the same decrease in neural activity, depressive-like behavior increased in PAE but not C females.

#### Amygdala

The amygdala is well known for its role in emotional processing and regulation of the neuroendocrine stress response (Ulrich-Lai and Herman, 2009; Myers et al., 2012), and dysregulation of the amygdala has been associated with major depression (Nestler et al., 2002; Ressler and Mayberg, 2007; Drevets et al., 2008; Krishnan and Nestler, 2010). Sensory information from thalamic and cortical inputs are initially processed and integrated in the lateral nucleus (LeDoux et al., 1990; Li et al., 1996; McDonald, 1998; Szinyei et al., 2000), which then transfers integrated information, either directly or relayed through the basal nucleus (Pitkänen et al., 1995; Savander et al., 1995), to the central nucleus—the major output nucleus of the amygdala. In the present study, we found no effects of PAE in females, but in males, PAE reduced *c-fos* mRNA expression in the lateral and central nuclei of the amygdala. Clinical studies find that in individuals with depression, the amygdala is often hyperactive in response to an emotional stimulus (Sheline et al., 2001; Drevets et al., 2002, 2008; Carlson et al., 2006). Therefore, a decrease in immobility in the FST would be expected to accompany the PAE-induced decrease in neural activity; instead, we found that PAE males were not different from controls in the FST, regardless of prior CUS. Although the changes in neural activation in the amygdala do not appear to associate with our behavioral findings, these findings suggest that the amygdala may receive (lateral nucleus) and relay (central nucleus) information differently in PAE compared to control males, which may subsequently alter their response to environmental cues.

#### Hippocampal formation

The hippocampal formation plays a major role in learning and memory, as well as in emotional and stress regulation (Fanselow and Dong, 2010), and clinically, has been suggested to play a role in depression (Videbech and Ravnkilde, 2004; MacQueen and Frodl, 2011). We found no differences among prenatal treatments for females, but in males, PAE rats had higher *c-fos* mRNA expression in the CA1 subregion relative to controls in response to delayed testing. Furthermore, inspection of Figure 6C suggests that the higher *c-fos* expression in PAE males may be due to the small differential decrease in CA1 activity in C and PAE males in the CUS-14 compared to the non-CUS condition, which is consistent with previous findings in PAE compared to C males in CA1 activity following stress (Raineki et al., 2014). Although we did not observe behavioral differences in the FST between C and PAE males, given that CA1 is a major output site of the hippocampus, projecting to over 50 areas (Cenquizca and Swanson, 2007), it is possible that we have unmasked a potential deficit in PAE compared to C males in being able to appropriately modulate hippocampal activity following CUS exposure.

#### Paraventricular nucleus of the hypothalamus

The mpdPVN integrates stress-related input from, but not exclusive to, the mPFC, amygdala and hippocampal formation to mount an appropriate HPA response to stress (Adhikari et al., 2010; Myers et al., 2012). We found that both male and female PAE animals showed neural activity in the PVN comparable to controls regardless of CUS history. These findings are consistent with those from our previous study in which no differences in *c-fos* mRNA expression in the mpdPVN were found among prenatal treatment groups, regardless of CUS exposure (Raineki et al., 2014). However, PAE has previously been shown to increase neural activation of the PVN compared to that in controls in response to stressors such as footshock (Lee et al., 2000). It has been suggested that the magnitude and time course of activation may be stressor-dependent (Cullinan et al., 1995; Duncan et al., 1996). While we observed no differences among prenatal treatment groups following FST, regardless of CUS exposure, the possibility that the time course of activation following FST may be different from that of footshock, or that PAE may alter the pattern of activation cannot be ruled out. Further investigations are needed.

### Constrained principal component analysis (CPCA)

Extending our statistical analysis with CPCA allowed us to move beyond examination of individual brain areas and identify potential neural activation networks associated with interactive effects of PAE and CUS, on FST behavior. CPCA highlighted three important characteristics of our data. First, we found different networks for males and females. Importantly, while FST behaviors did not load with either brain network in males, FST behaviors loaded along with Cg1 in the first component in females. These CPCA findings suggest that PAE and CUS may have greater impacts on FST behavior in females, and emphasize that interactive effects of PAE and CUS are sexually dimorphic, underscoring the importance of examining sex differences in outcome.

Second, CPCA revealed altered activation of brain subregions (e.g., mPFC, hippocampal formation, and mpdPVN) that the univariate analyses did not readily expose. Furthermore, it showed that the amygdala was not recruited in any of the networks, suggesting that it is not uniquely affected by our experimental conditions in either males or females. Identification of these effects was possible because the external analysis step of CPCA essentially removed irrelevant noise, permitting us to focus on the portion of the overall variance that is predictable from the interaction between prenatal treatment and CUS condition.

Third, the neural activation patterns for each sex were strikingly different among C, PF, and PAE animals. In males, PAE and C showed opposite patterns of activation for the *Medial prefrontal cortex* but not the *Hippocampal formation* network, suggesting that the functionality of this network is fundamentally altered by PAE. Moreover, CUS exposure differentially altered the activity of this network in PAE and C males. Given the role of the mPFC in cognitive processing and reappraisal/suppression of negative emotion (Koenigs and Grafman, 2009), the differential activation of the *Medial prefrontal cortex* network in PAE and C males following CUS may confer a predisposition to develop symptoms of stress-related disorders, such as depression, beyond the “behavioral despair” measured in the present study.

Interestingly, PAE females differed from PF females in their pattern of activation of the *FST behavior* + *Cg1* network, suggesting that the effects of PAE are not simply due to the effects of reduced food intake and the concomitant effects of that procedure, but are unique to alcohol exposure. PAE females also differed from C females for the *Hippocampal formation* network, suggesting that the functionality of this network is fundamentally altered by PAE. Furthermore, CUS exposure differentially altered the activity of both networks in PAE, PF, and C females depending on whether testing occurred immediately or following a delay. Overall, our findings indicate that PAE females engage neural strategies different from those of both PF and C animals. That differential effects of CUS on PAE, PF, and C animals depended on the timing of testing suggests that certain effects may take time to appear, while some effects may be transient.

### Pair-feeding

In general, we found no effects of PF in the individual behavioral and brain measures. However, CPCA revealed striking differences in the pattern of neural network activation in PF compared to both controls and PAE. Opposing patterns of *Hippocampal formation* network activation were found for PF and C males, opposing patterns of *FST behavior* + *Cg1* network activation were found for PF and PAE females, and opposing patterns of *PVN* network activation were found for PF and C females. The PF group, which receives a reduced ration of a nutritionally optimal diet matched in amount to that of an alcohol-consuming partner (Weinberg, 1984, 1985), is used to control for the effects of typical reduction in consumption of diets containing alcohol. However, as pair-feeding cannot control for the nutritional impacts of alcohol, such as effects on nutrients absorption and utilization, it is an imperfect control group. Additionally, these animals likely experience a component of prenatal stress because their daily rations, which are less than would be presented if given *ad libitum* access, are typically consumed within hours of food presentation, resulting in food deprivation and likely hunger until feeding the next day. Therefore, the behavioral, physiological, and neurological effects of PF on offspring represent, at least partially, the combined prenatal impact of mild stress and a reduced food ration. Interestingly, we previously found that PAE and PF dams differed in their maternal behavior, including nursing frequency, as well as off-nest, self-directed, and negative behaviors, but no differences in arched-back nursing, and licking and grooming behavior (Workman et al., 2015). Thus, it is possible that some of the differences in PAE and PF offspring may be mediated by different mother-infant interactions (Workman et al., 2015). Our CPCA findings that PF animals were in many ways different from both their C and, more importantly, PAE counterparts, provide support that PF is a treatment in itself and PAE effects on behavior and brain are not simply due to the effects of reduced food intake, but unique to the effects of alcohol exposure during prenatal development.

### Summary and implications

The present results build on and expand existing knowledge of the impact of PAE on neural regulation of behavior, stress and emotion that may underlie increased vulnerability to psychopathologies such as depression. The sex-dependent PAE-induced changes identified may also underlie the stress dysregulation that is observed following PAE, which in itself can contribute to psychopathologies. We also identified several effects of CUS that may have a delayed onset, underscoring the value of studying time-dependent effects of CUS in providing a more comprehensive and clinically relevant understanding of the effects of chronic stress. Importantly, the use of CPCA allowed us to go beyond simple assessments of changes in individual brain areas, and proved a powerful tool for identifying sex-dependent changes in functional networks in PAE animals compared to controls, and the differential recruitment of these networks among prenatal treatment groups following CUS exposure, thus allowing us to see a bigger picture in addition to discrete examinations of individual brain areas. By examining PAE- and CUS-induced differences in individual measures of depressive-like behavior and neural activity along with a network approach, we provide a more complete representation of the adverse effects of the interaction between PAE and CUS on brain and behavioral outcome, as well as insight into the pathophysiology of depression. Taken together, our findings suggest that PAE, in interaction with later life stress, results in sexually-dimorphic dysregulation of the neurocircuitry that subserves behavioral, emotional and stress regulation. In turn, this dysregulation may ultimately contribute to an increased vulnerability to psychopathologies, such as depression, that are often observed in individuals prenatally exposed to alcohol.

## Author contributions

VL, CR, LE, and JW designed the experiment; VL, CR, LT, and LE collected the data; VL analyzed the data, with the assistance of TW for CPCA; VL and JW wrote the manuscript. All authors edited the manuscript.

### Conflict of interest statement

The authors declare that the research was conducted in the absence of any commercial or financial relationships that could be construed as a potential conflict of interest.
